# Determination of cadmium and lead in tomato (*Solanum lycopersicum*) and lettuce (*Lactuca sativa*) consumed in Quito, Ecuador

**DOI:** 10.1016/j.toxrep.2020.07.008

**Published:** 2020-07-23

**Authors:** David Romero-Estévez, Gabriela S. Yánez-Jácome, Karina Simbaña-Farinango, Pamela Y. Vélez-Terreros, Hugo Navarrete

**Affiliations:** a Centro de Estudios Aplicados en Química CESAQ-PUCE, Pontificia Universidad Católica del Ecuador, Av. 12 de Octubre 1076 y Roca, Quito, 17012184, Ecuador; b Herbario QCA, Escuela de Ciencias Biológicas, Pontificia Universidad Católica del Ecuador, Quito. Av. 12 de Octubre 1076 y Roca, Quito, 17012184, Ecuador

**Keywords:** AOAC, Association of Official Analytical Chemists, CXS, General Standard for Contaminants and Toxins in Food and Feed Codex, DMQ, Metropolitan District of Quito, DNA, deoxyribonucleic acid, EPA, Environmental Protection Agency, FAO, Food and Agriculture Organization, HQ, hazard quotients, INEN, Ecuadorian Standardization Service, NTE, Ecuadorian Technical Standard, RSD, relative standard deviation, TM, trace metal, WHO, World Health Organization, Atomic absorption spectrophotometry, Fairs, Organic crops, Markets, Nonorganic crops, Trace metals

## Abstract

Vegetables are one of the most important components in the human diet, but despite their multiple nutritional components, studies have demonstrated the presence of trace metals in their edible parts. In Ecuador, two of the most consumed crops are tomato (*Solanum lycopersicum*) and lettuce (*Lactuca sativa*). The importance of these two crops in the Ecuadorian diet, especially in large and touristic locations like the Metropolitan District of Quito, implies food safety-related concerns for locals and visitors. However, no previous studies have quantified the cadmium and lead levels in these two vegetables using samples from Quito markets. Thus, the aim of this study was to determine the cadmium and lead content in both tomato and lettuce products from main nonorganic and organic markets in Quito using a graphite furnace atomic absorption spectrophotometer. The results showed that the cadmium levels were lower than 0.058 in tomatoes and 0.034 mg/kg in lettuce, which are under the respective threshold values (0.100 and 0.200 mg/kg). Regarding lead, levels lower than 0.066 mg/kg were detected in lettuce, which did not exceed the CXS 193–1995 threshold value, while levels in tomatoes were near or exceeded the threshold value (0.100 mg/kg) from four markets (0.209, 0.162, 0.110, 0.099 mg/kg), suggesting a possible risk from tomato consumption. In addition, most vegetables marketed as organic had higher metal content than those coming from nonorganic markets. Based on these results, local health and commercial control authorities should monitor contaminants in food products sold in Quito and other places in Ecuador to ensure their safety.

## Highlights

• Cadmium content was lower than 0.100 mg/kg (tomato) and 0.200 mg/kg (lettuce).
• Lead content above or close to 0.100 mg/kg was found in 25 % of tomato samples.
• Organic products had similar lead and cadmium content as nonorganic ones.

## Graphical abstract

In most of the samples, each individual metal content does not overpass the FAO/WHO threshold limits, nevertheless 25 % of the tomato samples were near or exceeded the Pb threshold value. The total metals content suggests that there is an important contamination in tomatoes and lettuces commercialized in the markets from Central and South zones of the DMQ. In addition, the organic products analyzed have an alarming metal content that should be considered.

**Figure:**
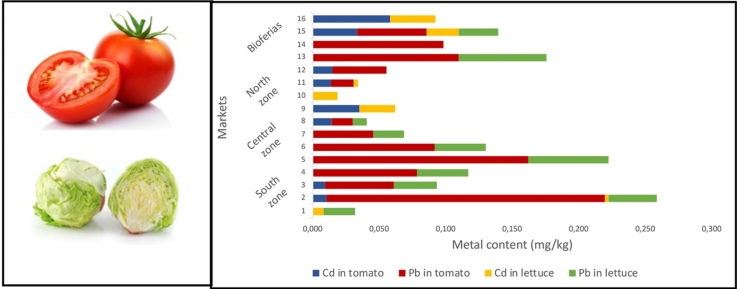

## 1 Introduction

Trace metals (TMs) constitute one group of known hazardous substances; in some cases, these elements are naturally present in soils [1], as they originate from the erosion of rocks or volcanic activity. Additionally, anthropogenic activities like mineral processing; chemical, metallurgic, petrochemical, and textile industries; and fuel combustion, among others [2], have increased TM concentrations. In both cases, these contaminants are readily available for intake by plants because they are present mostly in the soil surface, along with plant nutrients [1,3].

There is worldwide concern about some metals, such as cadmium (Cd), iron (Fe), and zinc (Zn), because they have the capacity to translocate into plant shoots; other metals like arsenic (As), chromium (Cr), and lead (Pb) bioaccumulate in most plant organs: roots, stem, leaves, and fruits [1]. In addition, TMs are nonbiodegradable, and they can move through food webs to ultimately be consumed by humans, which may result in various health risks due to their acute or chronic toxicity [[4], [5], [6]]. This concern has led researchers to test and apply different methodologies for sample preparation and metal quantification in food samples, including acid calcination [7], microwave-assisted acid digestion [[8], [9], [10]], and the use of absorbents and nanoparticles to extract TMs before quantification [[11], [12], [13]].

In crop cultivation, organic waste is commonly used as a source of nutrients, but long-term application can increase TM concentrations in soils [14]. In cultivable soils, Cd presence is assumed to occur from the soils’ own natural volcanic composition, but it can also follow when some fertilizers (e.g., phosphorous derivatives) are used. Once consumed, this metal is retained in the human body, particularly in the kidneys, producing highly toxic effects and increasing the risk of kidney failure and cancer [15]. For nonsmoking populations, food consumption is the main source of Cd exposure [3]. Pb is another harmful TM that can damage the nervous, skeletal, circulatory, enzymatic, endocrine, and immune systems [5].

The presence of metals in vegetables is influenced by many factors, principally by the crop species and its metabolism, and by others such as the soil’s initial concentration of contaminants, pH, organic matter availability, and presence of other ions and molecules [16,17]. The kinetics of TMs’ uptake depends on the mechanisms of their ion toxicity, including the blocking of functional groups in biomolecules and the replacing of essential metal ions in biomolecules [18]. Crops that are located close to vehicular traffic and factories also have an increased likelihood of TM presence [19].

Considering that vegetables are an important dietary source of essential nutrients, especially in terms of their high content of protein, vitamins, minerals, and fibers as well as beneficial antioxidant effects [20], their consumption is generally recommended [21]. Nevertheless, vegetables may also contain elevated concentrations of TMs due to high transfer from the soil to the harvested crop [1]. Further, since the early 20th century, human exposure to TMs from natural food consumption has increased because of their intensified use in industrial processes and products [5].

To protect food safety and human health, national and international organizations have established maximum permissible levels of TMs in food meant for human consumption [3,21]. One such regulation is the General Standard for Contaminants and Toxins in Food and Feed Codex (CXS) 193–1995 [22]. In addition, the Environmental Protection Agency (EPA) proposed the use of hazard quotients (HQs), which help evaluate the potential exposure status of a population as related to their alimentary habits [23,24]. The HQs have been extensively used to assess human health risk from vegetable consumption [4,5].

In Ecuador, two of the most consumed crops in the population’s daily diet are tomato (*Solanum lycopersicum*) and lettuce (*Lactuca sativa*), which are frequently used in salads and sandwiches. Each Ecuadorian consumes on average four kilos of tomatoes per year [25], and lettuce demand and consumption are high because of its use in both traditional and gourmet recipes [26].

There are no local-context studies that have determined Cd and Pb content in tomato and lettuce sold in the main markets in the Metropolitan District of Quito (DMQ, for its name in Spanish). For this reason, the present study aims to determine the Cd and Pb concentrations in tomato and lettuce samples from organic and nonorganic markets in the DMQ, Ecuador, to quantify the concentration of these TMs and evaluate compliance with the CXS 193–1995 international standard [22].

## 2 Materials and methods

### 2.1 Study location

The DMQ was selected for the current study as it is the most densely populated city in Ecuador, with a population of 2,735,987 [27]. It is the country’s capital and one of the most touristic destinations of the region. In addition, the DMQ and its surroundings include different industrial and commercial enterprises as well as relatively heavy traffic around and within the city, which are potential pollution sources. At the same time, large-scale vegetable production is conducted near this area, principally to supply the main city markets.

The climate in Quito varies daily but remains relatively steady throughout the year; the weather ranges from warm, sunny days to cold and windy conditions mixed with rain. The average annual temperature in Quito is 18 °C with an annual low temperature around 7 °C and an annual high temperature around 25 °C [28].

### 2.2 Sampling

All formal and registered nonorganic markets within the DMQ were considered for sampling, using information from the Direction of Markets, Fairs, and Platforms from the District Trade Coordination Agency of the DMQ municipality. Supermarkets were excluded because they have a limited number of approved vegetable providers, thus causing reduced variety in the samples. Additionally, organic markets (bioferias) were also sampled as another subgroup using information from the Economic Promotion Agency (CONQUITO) of the DMQ municipality.

The nonorganic markets were marked on a city map, which was divided into North, Central, and South zones; then, considering that there are four times as fewer bioferias than nonorganic markets, these organic markets were considered another group for this study.

Sixty-six markets were identified: 53 nonorganic markets, mixed markets, submarkets, and fairs and 13 organic bioferias.

Once located on the map and grouped by location/type, the representative amount of markets for the sample was calculated using the whole square root of the total number of markets per zone, shown in Table 1.

**Table 1:** Number of sampled locations for each market subgroup.

Subgroup	Total number of markets	Number of sampled markets
North zone	17	4
Central zone	18	4
South zone	18	4
Bioferias	13	4
**Total**	66	16

The following markets were randomly selected:

• North zone: “Bellavista” mixed market, “La Carolina” mixed market, “Rumiñahui” mixed market, and “San Antonio” mixed market.
• Central zone: “América” mixed market, “Arenas” market, “Jaime Roldós” fair, and “Toctiuco” market.
• South zone: “El Calzado” submarket, “Conocoto” mixed market, “Guamaní” fair, and “El Tingo” fair.
• Bioferias: “Conquito,” “La Floresta,” “Quito Tenis,” and “Quitumbe.”

In every market, the Ecuadorian Standardization Service’s (INEN) Ecuadorian Technical Standard (NTE) 1750:1994 was applied in the selection of samples [29], considering that every vendor had fewer than 100 kg of each product. A total of two kg of tomatoes and 10 units of lettuce were randomly selected from one vendor, collecting from five different vendors in each market. A total of 80 tomato and 80 lettuce samples from five vendors from each of the 16 markets were purchased and transferred to the Centro de Estudios Aplicados en Químca (CESAQ) laboratory. Then, all sample products were refrigerated at 4 ± 2 °C until their preparation.

During the sampling process, the number of vendors from whom samples were taken was selected according to the total number of vendors inside each market. In some cases, (Bellavista, Arenas, Guamaní, and Conquito), when there were less than five vendors, one vendor was considered as two or more lots.

### 2.3 Analysis of samples

The sample preparation and quantification methods were done using heat drying, microwave-assisted acid digestion, and graphite furnace atomic absorption spectrophotometry, taking as reference the methods described in Romero-Estévez et al. [10].

Initially, all samples were washed with high-quality reagent water (resistivity: 18.2 MΩ cm) to eliminate impurities. For both tomatoes and lettuce, the five samples from the same market were mashed and homogenized to obtain a composite sample (16 total composite samples for each vegetable). The water content of the composite samples was determined using a humidity analyzer (Mettler Toledo, HB43-S, Greifensee, Switzerland). Then, the composite samples were dried for 48 h at 60 °C in a Memmert UM 500 stove (Schwabach, Germany).

One (1.0000) gram of each composite sample was weighed in Teflon vials, where 5 mL of 70 % nitric acid (Fisher Chemical, Certified ACS plus, CAS# 7697-37-2) and 3 mL of 30 % hydrogen peroxide (Fisher Chemical, Certified ACS plus, CAS# 7722-81-1) were gradually added. Acid digestion was performed using a MARS 6 microwave (CEM, Matthews, NC, USA).

For the quantification, the obtained digestions were filtered. Then, Cd and Pb content were determined using an absorption spectrophotometer coupled to a graphite furnace (HGA 900 and AAnalys 400, Perkin Elmer Inc., Waltham, MA, USA), using the temperature programming described in Table 2. Calibration curves were prepared using four different concentration levels of dilutions of certified reference materials of 0.5, 1.0, 2.0, and 4.0 μg/mL for Cd (Inorganic Ventures, 1000 μg/mL, Certified Standard, CAS # 7697-37-2) and 5.0, 10.0, 20.0, and 40.0 μg/mL for Pb (Inorganic Ventures, 1000 μg/mL, Certified Standard, CAS # 7697-37-2). A linear regression coefficient (R^2^) of a minimum of 0.99 was expected to demonstrate linear adjustment between concentration and absorbance. All the standards of the calibration curves, samples, and blanks were prepared using analytical grade reagents and high-quality reagent water. The results are presented in mg/kg of dry and fresh weight. Matrix modifiers were also used: for Cd analysis, a mixture of 0.015 mg Pd (Inorganic Ventures, Matrix modifier, CAS # 7697-37-2) and 0.01 mg Mg(NO_3_)_2_; for Pb, 0.2 mg NH_4_H_2_PO_4_ (Inorganic Ventures, Matrix modifier, CAS # 7697-38-2).

**Table 2:** Temperature programming of the graphite furnace used to analyze the Cd and Pb in the samples.

Temperature (°C)		Ramp Time (s)	Hold Time (s)
Cadmium	Lead		
110	110	1	30
130	130	15	30
850	700	10	20
1650	1800	0	5
2450	2450	1	3

All composite samples were analyzed in duplicate, and reagent blanks and fortifications in known concentrations (0.050 mg/kg for Cd and 0.250 mg/kg for Pb) prepared from certified reference materials of approximately 1000 mg/mL were added to original non-fortified samples and used as quality control. The relative standard deviation (RSD) and the accuracy as relative recovery rates of fortifications were also evaluated using the criteria established in the Guidelines for Single Laboratory Validation of Chemical Methods for Dietary Supplements and Botanicals of the Association of Official Analytical Chemists (AOAC) [30]: precision of 16.0 % for repeatability and recoveries between 75.0 % and 120.0 % for accuracy. In addition, a certified reference material of tomato leaves 1573a from the National Institute of Standards and Technology was used as an additional quality control.

The obtained results were compared with the CXS 193–1995 [22]. The corresponding threshold value in fresh weight for tomato is 0.100 mg/kg for both Cd and Pb; for lettuce, the values corresponding to fresh weight are 0.200 mg/kg for Cd and 0.300 mg/kg for Pb [22].

## 3 Results and discussion

All the calibration curves presented R^2^ values higher than 0.99. The limits of detection (LOD) and the limits of quantification (LOQ) were calculated by using low-level concentration fortifications for each metal. The obtained LOD values were 0.010 and 0.150 μg/kg for Cd and Pb, respectively, and the obtained LOQ values were 0.050 and 0.250 mg/kg for Cd and Pb, respectively.

As shown in Table 3, Cd results in tomatoes indicated that the 16 composite samples had concentrations lower than the CXS 193–1995 threshold values (0.100 mg/kg, fresh weight); the highest values were found in bioferias and North zone markets. In addition, three markets (two bioferias and one from the Central zone) had non-detectable results.

**Table 3:** Cadmium results in tomato and lettuce from 16 of the principal markets in Quito, Ecuador.

Market	Zone	TOMATO (*Solanum lycopersicum*)				LETTUCE (*Lactuca sativa*)			
		Cadmium content in samples [mg/kg] (dry weight)	Cadmium content in samples [mg/kg] (fresh weight)	RSD [%]	Relative recovery [%]	Cadmium content in samples [mg/kg] (dry weight)	Cadmium content in samples [mg/kg] (fresh weight)	RSD [%]	Relative recovery [%]
1	South	<0.050	<LOQ	--	84.2 %	0.166	0.008	2.4%	82.1 %
2	South	0.115	0.010	5.5%	102.0 %	0.054	0.002	2.2%	119.3 %
3	South	0.100	0.009	8.9%	104.7 %	<0.050	<LOQ	--	81.4 %
4	South	<0.050	<LOQ	--	115.8 %	ND	ND	--	94.5 %
5	Central	ND	ND	--	87.2 %	<0.050	<LOQ	--	80.9 %
6	Central	<0.050	<LOQ	--	79.0 %	<0.050	<LOQ	--	85.7 %
7	Central	<0.050	<LOQ	--	119.3 %	ND	ND	--	84.2 %
8	Central	0.147	0.014	10.7%	112.7 %	<0.050	<LOQ	--	91.2 %
9	North	0.378	0.035	3.8%	78.4 %	0.565	0.027	2.4 %	114.5 %
10	North	0.142	<LOQ	--	113.8 %	0.245	0.019	1.6 %	104.2 %
11	North	0.142	0.014	6.3%	84.6 %	0.094	0.003	14.7 %	76.5 %
12	North	0.157	0.015	8.5%	97.9 %	<0.050	<LOQ	--	103.7 %
13	Bioferia	ND	ND	--	78.7 %	ND	ND	--	77.3 %
14	Bioferia	ND	ND	--	117.6 %	ND	ND	--	91.7 %
15	Bioferia	0.350	0.033	3.5%	116.5 %	0.450	0.024	2.2%	119.2 %
16	Bioferia	0.660	0.058	0.3%	105.0 %	0.431	0.034	1.8%	118.9 %
Threshold values^a^			0.100				0.200		
Acceptance limits^b^				16.0 %	75.0–120.0 %			16.0 %	75.0–120.0 %

RSD: Relative standard deviation; <LOQ: lower than the limit of quantification; ND: Not detectable.

a FAO/WHO. General Standard for Contaminants and Toxins in Food and Feed CXS 193–1995 (Revision 2019).

b AOAC. Guidelines for Single Laboratory Validation of Chemical Methods for Dietary Supplements and Botanicals.

For lettuce, the 16 markets also showed results lower than the CXS 193–1995 threshold values (0.200 mg/kg, fresh weight). The highest results for Cd were found in the same bioferias that had the highest Cd levels in tomatoes and markets from the North zone. Four markets showed no detectable results (two bioferias, one from the Central and one from the South zone).

The Pb quantification results (Table 4) demonstrated that for tomatoes, 4 of the 16 markets sampled (25 %) had Pb content near or exceeding the CXS 193–1995 threshold values (0.100 mg/kg, fresh weight). Two markets had Pb in non-detectable concentrations, and the highest values were found in markets from the South zone and bioferias. In the case of lettuce, Pb results did not exceed the CXS 193–1995 threshold values (0.300 mg/kg, fresh weight), and the highest values were found in markets from the Central zone and one bioferia.

**Table 4:** Lead results in tomato and lettuce from 16 of the principal markets in Quito, Ecuador.

Market	Zone	TOMATO (*Solanum lycopersicum*)				LETTUCE (*Lactuca sativa*)			
		Lead content in samples [mg/kg] (dry weight)	Lead content in samples [mg/kg] (fresh weight)	RSD [%]	Relative recovery [%]	Lead content in samples [mg/kg] (dry weight)	Lead content in samples [mg/kg] (fresh weight)	RSD [%]	Relative recovery [%]
1	South	<0.250	<LOQ	--	93.9 %	0.499	0.024	2.3%	87.7 %
2	South	2.327	0.209	0.5%	115.6 %	0.787	0.036	1.3%	105.2 %
3	South	0.544	0.052	1.0%	103.9 %	0.808	0.032	1.7%	116.1 %
4	South	0.810	0.079	0.3%	88.0 %	1.005	0.038	1.5%	118.6 %
5	Central	1.637	0.162	0.2%	116.1 %	1.182	0.060	0.2%	82.3 %
6	Central	0.994	0.091	1.6%	90.6 %	0.996	0.039	1.5%	102.8 %
7	Central	0.472	0.045	2.9 %	95.5 %	0.636	0.024	0.5%	94.3 %
8	Central	<0.250	<LOQ	--	86.1 %	0.254	0.010	2.5%	93.2 %
9	North	ND	ND	--	103.6 %	ND	ND	--	112.6 %
10	North	<0.250	<LOQ	--	87.1 %	<0.250	<LOQ	--	103.8 %
11	North	<0.250	<LOQ	--	102.2 %	<0.250	<LOQ	--	115.5 %
12	North	0.437	0.041	2.7%	103.3 %	<0.250	<LOQ	--	108.4 %
13	Bioferia	1.018	0.110	1.3%	118.0 %	1.077	0.066	1.1%	99.2 %
14	Bioferia	0.938	0.099	0.7%	93.0 %	<0.250	<LOQ	--	118.1 %
15	Bioferia	0.526	0.053	1.3%	94.8 %	0.557	0.029	1.6%	81.3 %
16	Bioferia	ND	ND	--	116.8 %	<0.250	<LOQ	--	113.9 %
**Threshold values**^a^			0.100				0.300		
**Acceptance limits**^b^				16.0 %	75.0–120.0 %			16.0 %	75.0–120.0 %

RSD: Relative standard deviation; <LOQ: lower than the limit of quantification; ND: Not detectable.

a FAO/WHO. General Standard for Contaminants and Toxins in Food and Feed CXS 193–1995 (Revision 2019).

b AOAC. Guidelines for Single Laboratory Validation of Chemical Methods for Dietary Supplements and Botanicals.

As mentioned, for tomatoes, the Pb concentration in 25 % of the samples was near or exceeded the corresponding suggested threshold value. These results could indicate an important source of Pb contamination and a possible hazard from the consumption of these and similar products, especially if consumption is high and frequent, which could increase the probability of health problems associated with Pb toxicity. The contamination level of food and the effectiveness of actions to reduce contamination should be assessed by monitoring, survey programs, and more specialized research programs where necessary [22]. In this sense, local health and commercial control authorities should monitor the contaminants in food products sold in the DMQ and other places in Ecuador to ensure their safety.

The quality control results were consistent with AOAC criteria [30]. For precision, considering the RSD values, the highest were 14.7 % and 2.9 % for Cd and Pb, respectively. The fortification relative recoveries were between 77.3 % and 119.3 % for Cd and between 81.3 % and 118.6 % for Pb. The recoveries of the certified reference material used were between 87.2 % and 98.1 % for Cd.

**Fig. 1:**
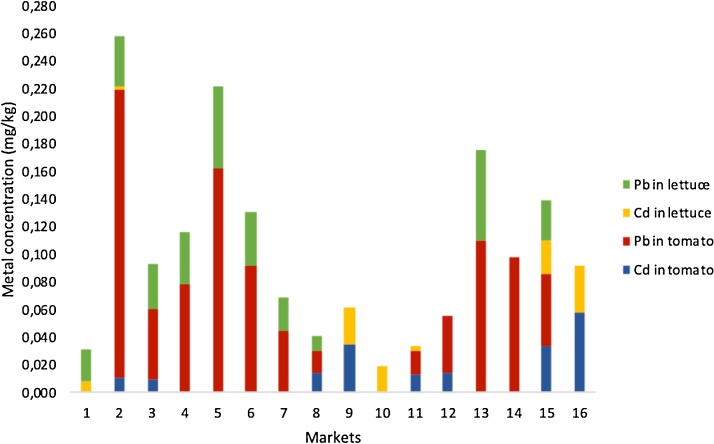
Total metal content (cadmium [Cd] and lead [Pb]) in tomatoes and lettuce from 16 principal markets in Quito, Ecuador.

Considering the total amount of Cd and Pb in the tomato and lettuce samples, the markets from the North zone (9–12) had the lowest concentration of metals. The highest metal content was found in markets 2, 5, and 13, corresponding to the South and Central zones and one bioferia, respectively (Fig. 1). This result shows that the metal content is unrelated to market location; rather, markets in the same zones had different results, which confirms that the tomatoes and lettuce sold differed in terms of origin.

Various studies related to these toxic metals’ content in tomatoes and lettuce have been conducted in products from local markets in Ecuador and other countries. Table 5 shows the present study’s results compared to those obtained by other studies.

**Table 5:** Ranges of cadmium and lead concentrations from studies conducted in different countries.

Country	Cadmium content (mg/kg)		Lead content (mg/kg)		Reference
	Tomato	Lettuce	Tomato	Lettuce	
Ecuador (Quito)	0.009–0.058	0.002–0.034	0.041–0.209	0.010–0.066	The present study
	<0.050–0.660*	<0.050–0.565*	<0.250–2.327*	<0.250–1.182*	
Ecuador (Quito)	--	0.008–0.019	--	0.0011–0.027	[31]
Ecuador (Quito)	0.008–0.024	0.004–0.005	0.001–0.004	0.031–0.273	[32]
Croatia (Varaždin)	--	0.97–1.52*	--	0.82–2.22*	[1]
Argelia	--	26.99*	--	--	[5]
Chile (metropolitan region)	--	--	<0.014–0.057	<0.143–0.208	[33]
Brazil (Belo Horizonte)	--	--	0.009*	0.037*	[19]
Brazil (Pernambuco)	--	--	<0.01	<0.06	[34]
Spain (Barcelona)	--	--	<0.005	0.0080–0.0244	[35]
Spain (Valencia)	0.0055	0.0098	0	0.0038	[45]
India (Amravati)	0.2*	5.5*	--	--	[36]
India (Nashik)	0.90*	2.20*	--	--	[37]
India (Andhra Pradesh)	0.0193	--	0.3	--	[46]
USA (Florida) organic products	0.00093	0.00495	0.01450	0.0120	[38]
USA (Florida) nonorganic products	0.0043	0.00772	0.00487	0.0253	
Jamaica	0.266	--	0.021	--	[44]

* Dry weight.

In Carrillo Quezada’s [31] study, lettuce samples from 30 DMQ markets showed slightly lower results than those from the present study for both Cd and Pb. Further, in a later study by Velasquez Paredes [32], in tomato and lettuce samples collected from two of the DMQ’s main markets, the Cd results were also lower than the present study’s in both vegetables. However, Velasquez Paredes [32] found Pb content in lettuce samples that was approximately four times higher than that found in the present study, whereas for tomatoes, the present study’s Pb results were approximately 50 times higher. This heterogeneity in TM levels strongly suggests that determining a TM concentration trend is not possible since even within the same market, TM levels vary by vegetable and vegetable origin; therefore, frequent control of these contaminants in vegetables is needed.

The maximum Cd concentration obtained in tomatoes (0.660 mg/kg, dry weight) in the current study was three times higher than that reported by Mohod [36] from Amravati, India, but lower than that reported by Labhade [37] from Nashik, Murad Basha et al. [46] from Andhra Pradesh, India, and quite lower than that reported by Murad Basha et al. [44] from Jamaica. Further, the concentrations reported by Hadayat et al. [38] from Florida (USA) in both organic and nonorganic tomatoes were considerably lower than those from the present study, the same happened with the concentrations reported by Marín et al. [45] from Valencia. For Cd content in lettuce, the dry weight results obtained by the present study were largely lower than those reported in Croatia [1], Argelia [5], and India [37], but higher than the value assessed in Valencia [45]. This heterogeneity could be due to several external factors that could affect the TM content in natural products, such as the initial metal content in the crop soils and the environmental pollution near the cultivation sites.

The Pb results in lettuce samples for the current study showed values that were similar to those from Croatia [1], Brazil [19], Chile [33], and Spain [35]. In the case of tomatoes, the Pb concentrations obtained by the present study were higher than all the international studies in Table 5, except from the mining area of India (Andhra Pradesh) [46], which clearly demonstrate the possible toxicity of Ecuadorian tomatoes, especially those from the four markets where the samples exceeded the CDX 193-95 threshold values [22]. A study conducted by Silva et al. [39] demonstrated that even with low Pb concentrations, genotoxic effects in plants occur, as proven by the increase in the number of micronucleated cells and of DNA fragmentation.

When comparing the results between nonorganic and organic crops (bioferias), considering the values from the present study and those reported by Hadayat et al. [38], the metal content is completely different. Hadayat et al. [38] describe that in the analyses of organic market products, the TM concentration was lower than that found in nonorganic crops; the authors suggest this is due to the non-use of metal-based pesticides. In the present study, the samples from organic markets (bioferias) had Cd and Pb content in tomatoes and lettuce greater than that from the other three market groups selling nonorganic products. Further, the Cd content found by the present study and the results of Hadayat et al. [38] are contradictory. There has been a significant increase in consumer demand for organic products, which are marketed as such because of the relatively low use of chemicals in their cultivation and less processing. Nevertheless, a superior nutritional content and lower presence of toxins (TMs) that contribute to the safe consumption of these products have not yet been fully demonstrated. Proximal analyses of macro and micronutrients have shown a low nitrogen content in organic crops, but evidence in relation to other nutrients and contaminants is lacking [40].

Furthermore, the high levels of Cd and Pb in Ecuadorian vegetables may be because Ecuadorian soils are rich in some metals, including Cd and Pb [41,42]; this natural presence contributes to their incidence in crops. In addition, one general organic cultivation practice involves using the residue from the previous generation of plants as fertilizer; thus, all the metal content present in the leaves, stems, and other parts of the harvested plants is reincorporated into the soil, which replaces mineral nutrients for the next generation’s growth.

Moreover, even when urban agriculture plays an important role in cities’ sustainable food supply, the products need to be monitored and regulated because not only TMs but also other toxic substances from air pollution and soil contamination could be present [19]. Currently, industrial and motor vehicle emissions are one of the main sources of air pollution identified in urban sites, including big cities. These pollutants can be present in the particulate matter of air emissions and eventually be deposited in the soil and plants [34].

Adopting soilless cultivation systems that include good management practices can reduce crop soil contamination. In the last decade, the number of soilless cultivation systems in cities has increased because of the benefits they offer [35]. This distinct cultivation technique avoids the likelihood of the transference of contaminants and TMs from soils to vegetables, which is a long-term health and safety risk associated with urban agriculture. Dala-Paula et al. [19] found a significant positive correlation between Cd in lettuce and in soil; in the case of Pb, even when soil concentrations varied, its low mobility made no significant difference in the metal intake of plants. Contrary to this, in the present study, higher concentrations of Pb were found, and this could be related to the presence of this element in the atmosphere.

In cities like the DMQ, especially in locations in or near mountains, the natural presence (from volcanic eruptions, the weathering of rocks, and erosion) of contaminants like TMs can lead to high concentrations in soils. Adding to this natural presence are anthropogenic activities like irrigation; the use of fertilizers, pesticides, and sludge applications; industrial activities; solid waste disposal; mining; smelting; vehicular exhaust; and domestic and agricultural use of metals and metallic compounds [43]. All of these may necessitate the application of protocols for the cultivation, production, and transportation of agricultural products as well as education regarding possible sources of contamination and ways of mitigating the danger.

## 4 Conclusions

As no local studies have been conducted related to the content of Cd and Pb in tomatoes and lettuce sold in the main markets of all the different areas of the DMQ, this research is the first to determine the levels of these metals in products of daily consumption.

Although the results showed that in most of the cases, the TM concentrations were lower than the threshold values, their presence in agricultural products in Ecuador should still be considered. In the case of tomatoes and lettuce, TM contamination is not overtly related to agricultural and transportation processes; therefore, the TM content found in these products is assumed to be from soil contamination. This information needs to be considered by farmers so remediation techniques that reduce TM presence in their crops can be utilized. Further, Ecuadorian agricultural authorities should implement control mechanisms to ensure that food contaminants are within the maximum limits established in national and international regulations.

In addition, the controversy involving organic versus nonorganic agricultural products continues; nutritional information, toxicity levels, and even pricing of organic products are not fully supported by scientific evidence. The results of the present study show that in fact the organic products, which are considered “healthier” than nonorganic products, in most of the cases had higher concentrations of Cd and Pb.

In view of this study’s findings, more research should be conducted that not only analyzes the TM content in foodstuffs but also tracks pollution in the soil, water, and air. In addition, it is necessary to carry out studies focused on the differences between organic and nonorganic crop nutrients and the possible effect of contaminants like TMs on these nutrients.

## Funding

This research did not receive any specific grant from funding agencies in the public, commercial, or not-for-profit sectors.

## CRediT authorship contribution statement

**David Romero-Estévez:** Conceptualization, Methodology, Software, Validation, Formal analysis, Investigation, Data curation, Writing - original draft, Writing - review & editing, Visualization. **Gabriela S. Yánez-Jácome:** Writing - review & editing, Visualization. **Karina Simbaña-Farinango:** Formal analysis, Investigation. **Pamela Y. Vélez-Terreros:** Formal analysis, Investigation. **Hugo Navarrete:** Conceptualization, Resources, Supervision, Project administration, Funding acquisition.

## Declaration of Competing Interest

The authors declare that they have no known competing financial interests or personal relationships that could have appeared to influence the work reported in this paper.
